# Development of Internalizing Mental Health Symptoms from Early Childhood to Late Adolescence

**DOI:** 10.3390/ejihpe14080159

**Published:** 2024-08-18

**Authors:** Ioannis G. Katsantonis

**Affiliations:** Faculty of Education, University of Cambridge, Cambridge CB2 8PQ, UK; ik388@cam.ac.uk

**Keywords:** piecewise growth, internalizing mental health, children, adolescents, symptoms, growth curve model, Growing Up in Australia

## Abstract

Children’s mental health symptoms’ development can be characterized by both continuity and discontinuity. However, existing studies ignore the potential discontinuity in children’s internalizing symptoms’ development. Hence, the current study examines continuous and discontinuous developmental trajectories using representative data from a sample of 2792 children (49.10% females) from the Growing Up in Australia cohort assessed seven times (ages 4, 6, 8, 10, 12, 14, 16). Longitudinal measurement invariance analyses revealed that internalizing symptoms were comparable over time. Linear, quadratic, and piecewise latent growth curve models were deployed to estimate the trajectory of internalizing symptoms from early childhood to late adolescence. The analyses showed that internalizing symptoms were characterized by a quadratic-quadratic piecewise growth curve comprising two distinct phases of upward concave growth. Internalizing scores reduced steadily between ages 4 and 8 years but exhibited a slight upward curvature between ages 8 and 10 years. By age 14 years, the trajectory remained relatively stable but spiked between age 14 and 16 years. The two phases of internalizing symptoms’ development were largely unrelated. Overall, the study adds to the knowledge about the development of internalizing mental health from early childhood to late adolescence and highlights the need for additional support in late adolescence.

## 1. Introduction

Recent epidemiological studies have noted an increase in childhood emotional symptoms [1] and peer problems [2]. Emotional symptoms and peer problems belong to the wider spectrum of internalizing mental health symptoms referring to covert, self-focused signs of distress and excessively restrained reactions, characterized in research on children and adults as symptoms, conditions, or diagnoses [3]. These are symptoms of adverse mental health conditions and are also referred to in the literature as symptoms of mental health problems [4]. Given the reported increases in internalizing symptoms, it is important to examine the development of the internalizing mental health symptoms in the early life course to be able to gain deeper insights into sensitive periods for development. In particular, recent evidence suggests that mental health symptoms’ development [5,6] can be characterized by both periods of continuity and discontinuity, whereby earlier growth may hinder or exacerbate later developments. However, extant studies typically ignore tests for potential discontinuity in mental health symptoms’ development. Hence, the current study aims to provide robust representative evidence on the shape of the developmental trajectory of internalizing mental health symptoms from early childhood to late adolescence. 

### 1.1. Development and Discontinuity in Children’s Internalising Symptoms

In the general population, there is robust evidence indicating that many internalizing symptoms first manifest in childhood and adolescence [7]. Recent empirical studies have also reported that internalizing symptoms, such as emotional symptoms and peer problems [8], are reciprocally related in childhood and adolescence [9,10], suggesting that emotional symptoms drive increases in peer problems and vice versa. Because of this reciprocity, most studies examine potential developmental continuity in peer problems and emotional symptoms jointly using an appropriate broad index of internalizing symptoms, as will be shown below. Research studies have also highlighted that early internalizing symptoms can robustly predict internalizing scores later on as the children develop [11,12,13], but the strength of the regression coefficients is rather modest in most cases [10,12]. This suggests either the potential for other intervening factors or the existence of developmental discontinuity.

Regarding the development of internalizing symptoms, several extant empirical studies have examined the shape of the developmental trajectory. For example, a large-scale study with Irish children assessed three times at ages 3, 5, and 9 reported three linear trajectories called increasing, stable, low, and moderate [2]. Another longitudinal study in Britain with a sample of children at ages 3, 5, 7, and 11 also reported linear trajectories, with most children belonging to a stable low trajectory, followed by an improving trajectory and two deteriorating trajectories [14]. In contrast, a Swiss cohort study with children between ages 7 and 15 years showed that a linear and quadratic growth trajectory was better at describing the shape of internalizing symptoms’ development [15]. Although the above empirical studies have examined the trajectories of internalizing mental health symptoms across childhood and adolescence, these studies were concerned with developmental continuity in mental health development, whereby a single trajectory covered the whole developmental period. A limitation of that modeling choice is that it ignores the potential discontinuities that may occur, whereby there might be some distinct phases of development. 

It has long been recognized that the development of symptoms is usually characterized both by periods of continuous growth and periods of discontinuity [16] when critical life events and transitions take place [17,18]. However, few studies have to date examined the potential discontinuity in child and adolescent internalizing mental health symptoms’ development. For example, the study by Cohen et al. [5] found that both anxiety and depression (i.e., indicators of internalizing symptoms) were characterized by a linear-linear piecewise discontinuous development, whereby internalizing symptoms decreased up to age 12 and started increasing in early adolescence. Additionally, an important cross-cultural study by Rothenberg et al. [6] revealed that six cultural groups, namely Colombia, Italy/Naples, Italy/Rome, Jordan, U.S. White, and U.S. Black, had internalizing symptoms’ trajectories that were described by discontinuity via a linear-linear piecewise trajectory. In those cultural groups, internalizing scores decline between ages 8 and 10 years [6]. Furthermore, another longitudinal British study revealed that the development of internalizing symptoms could be characterized by a piecewise (i.e., stage-like; phasic) trajectory comprising one piece in childhood (ages 4–9) and another piece in adolescence (ages 11–16) [19]. Despite the above, one study examined the conflated the internalizing symptoms’ development with other symptoms [19], and the other two studies [5,6] did not examine the possibility that the piecewise trajectory might be better described by a quadratic-quadratic curve rather than a piecewise linear-linear curve.

### 1.2. Transitioning to Adolescence as a Turning Point for Internalising Symptoms’ Development

Specific transitions have been linked with developmental ‘turning points’ in psychopathology that create transient discontinuities in symptom expression [20]. The study of developmental discontinuity in internalizing symptoms is important because it can illustrate how childhood trajectories are linked with adolescent trajectories. An apt example of developmental discontinuity is an adolescent who is characterized by a mental health difficulty but grows up to be a healthy adult [21]. Hence, researchers should study in depth how early life experiences may reverse, eradicate, or mediate later experiences [16]. The working hypothesis is that adolescence will be found to be a crucial turning point for children’s internalizing mental health development. Researchers have indicated that adolescence is a critical stage where educational [22], social, and biological changes occur [23]. Specifically, it is expected that the onset of early adolescence (age 10 years) [24] will be a significant turning point for another discreet stage of internalizing mental health symptoms’ development. The reasons for this are several. First, puberty typically starts around 10 years old and coincides with the onset of early adolescence [24,25]. Second, early adolescence is characterized by growth not only in cognitive domains (e.g., abstract thinking) but also in socio-emotional domains (e.g., parent-child conflict, peer influences) [25]. Third, another major life event taking place in early adolescence (at least in developed countries) is the transition from primary to secondary school [22,26]. The latter is a usually overlooked aspect of child development that, nonetheless, has significant psychological implications [26]. Finally, anxiety disorders [27], major depressive disorder, and depression start between 11 and 14 years [28]. Hence, the current study investigates not only the shape of the developmental trajectory of internalizing symptoms but also the possibility of piecewise discontinuous growth by splitting the development before adolescence and after early adolescence. 

### 1.3. The Present Study

As mentioned above, the study of children’s internalizing symptoms’ development across early childhood and late adolescence is important in light of the reported increases in internalizing symptoms [29,30]. Most important, though, is the need to examine whether internalizing symptoms’ development is not only characterized by continuous growth over time but also by piecewise/discontinuous growth. Few studies have explored this possibility, some with non-representative samples [6], and some other studies have only estimated linear-linear piecewise combinations without considering quadratic piecewise growth [5,19]. Furthermore, past empirical studies examining growth in internalizing symptoms have dealt mostly with linear growth [2,14]; however, there is evidence that internalizing symptoms might be better described by nonlinear growth [1,15]. Therefore, more research is needed to investigate the optimal developmental trajectory of internalizing symptoms. Hence, the following research questions guide the present study:

RQ1: How do internalizing symptoms develop from early childhood to late adolescence?

RQ2: Is early adolescence a critical transitioning point for internalizing symptoms’ development?

Since linear growth might not be an accurate representation of internalizing symptoms’ development [1,15], it is hypothesized that the growth will be better represented by a quadratic growth function (H1). Given the theoretical support for both continuity and discontinuity in child development [17] and the empirical evidence on the piecewise growth of internalizing symptoms [5,6,19], it is hypothesized that the growth will be piecewise (H2). Because early adolescence is fraught with developmental changes [22], it is expected that the onset of early adolescence at age 10 years will be a critical turning point for internalizing symptoms’ development (H3).

## 2. Materials and Method

### 2.1. Participants and Dataset

The participants of the study comprised 2792 children who were assessed seven times (ages 4, 6, 8, 10, 12, 14, 16). The data come from the Growing Up In Australia cohort study, also known as the Longitudinal Study of Australian Children (LSAC) [31]. This is a longitudinal representative cohort study of the population of Australian children. The data were collected biennially between 2004 and 2016 (see [32]. The sampling design is a two-stage cluster-stratified random sampling [31]. Postcodes were first sampled, followed by a sampling of children within postcodes [33]. Stratification was utilized to ensure proportional representation from different geographical locations [31]. Children had an approximately equal probability of being selected to participate in the cohort study [31]. Given the above, the inferential and descriptive analyses need to account for this complex sampling design by applying the provided longitudinal sampling weights and adjusting the standard errors for the clustering and stratification [34]. This ensures that the results are representative of the wider Australian population of children at the target period.

The vast majority of the children in the current study spoke English as the main language at home (90.8%). The children were about evenly split into sex groups, with 49.1% females and 50.9% males. At age 10 years, most children were in primary school, and at age 12 years, most children were studying in secondary school. 

### 2.2. Measures

#### Internalizing Mental Health Symptoms

Internalizing mental health symptoms were measured using the parent-reported version of the Strengths and Difficulties Questionnaire—SDQ [8,35]. This means that the parent (in most cases, the biological mothers) reported on their child’s mental health symptoms. The summed composite score of the emotional symptoms and peer problems subscales creates an index of internalizing mental health symptoms for children aged 4 to 16 years old [8]. The use of a broader internalizing index is recommended for community samples [8], such as the current one. Sample items from the emotional symptoms’ subscale include “often complains of headaches” and “often unhappy or downhearted.” Sample items from the peer problems’ subscale include “rather solitary, tends to play alone,” and “has at least one good friend” (reversed). The items were scored using a 3-point scale ranging from 0, “not true,” to 2, “certainly true.” Higher scores indicate greater internalizing problems. The SDQ can predict some of the most common psychiatric disorders and has good specificity and sensitivity [36]. The SDQ has been administered to cohort studies also in the UK [37] and Ireland [2]. 

### 2.3. Covariates

Several time-invariant control variables were drawn from the first wave to fit conditional growth curve models to examine potential individual differences in children’s internalizing symptoms’ development [38]. These covariates were standard demographic variables such as the following. Children’s sex assigned at birth (female vs. male) was utilized as a time-invariant predictor at age 4 years, which is known to influence mental health symptoms’ development, with girls being more vulnerable [39]. Additionally, the responding parent’s income (in groups) (>95% mothers) was utilized as a time-invariant predictor at age 4 years. Another robust indicator of socioeconomic status considered here is the responding parent’s highest educational qualification [40,41], which was coded as university-level (bachelor’s, graduate degree, postgraduate degree) versus not-university-level (yes = 0). The parent’s marital status, recording whether the parent was married (yes = 1) or not (not married = 0), was also included in the conditional model since it can have adverse effects on children’s mental health symptoms [42]. The number of siblings living in the household is another important covariate considered here because research has shown that more siblings in the household are associated with fewer mental health symptoms [43,44]. Finally, whether the responding parent attended a religious service (yes = 1) was included because there is some evidence that religious attendance is associated with better mental health outcomes [45].

### 2.4. Statistical Analyses

**Figure 1:**
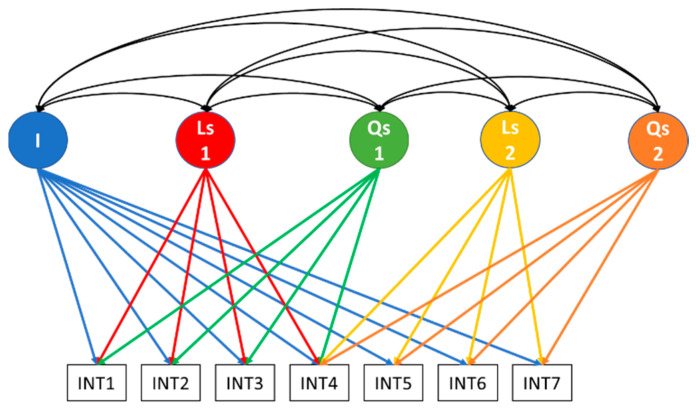
Quadratic-quadratic piecewise LGM with a knot at age 10 years; I: Intercept; Ls1: Linear slope 1 (ages 4 to 16); Qs1: Quadratic slope 1 (ages 4 to 16); Ls2: Linear slope 2 (ages 10 to 16); Qs2: Quadratic slope 2 (ages 10 to 16); INTt: Internalising symptoms at time t.

The statistical analyses began with testing of longitudinal measurement invariance (LMI) of emotional symptoms and peer problem scores across ages 4 to 16 years old. The single-group approach to LMI for ordered-categorical data was adopted to account for the longitudinal nature of the data [46]. Configural, metric, scalar, and strict invariance levels were tested [46]. Instead of imposing like-item residual correlations to account for shared method variance due to repeated measures [46], four indicator-specific method factors with their loadings fixed to 1 were specified to avoid negative residual variances [47]. Afterward, growth curve models (LGM) were fitted to the composite internalizing scores per time point to determine what trajectory shape (e.g., linear, non-linear, or piecewise) was a better approximation of the internalizing symptoms’ development. The LGM controls for the baseline symptoms (i.e., the intercept term) and measures (linear or non-linear) change in symptoms over time [38]. Specifically, linear, quadratic, and piecewise linear-linear, as well as piecewise quadratic-quadratic specifications were estimated. Linear growth is characterized by a straight line (trajectory), which increases or decreases by a fixed amount between time intervals [38]. Quadratic growth extends linear growth by adding another latent factor that captures the curvature or steepness of the increase or decrease [48]. Finally, the study moved beyond single-piece growth by estimating piecewise growth as well. The purpose of the piecewise LGM is to split the developmental trajectory into two stages/phases/pieces using a fixed transition point, also called a ‘developmental knot’ that ties together the two pieces/stages [49]. To specify a piecewise LGM, there is a common latent intercept factor that predicts all the repeated measures, a linear slope growth factor for the first piece, and an additional linear slope growth factor that predicts the repeated measures only after the fixed transition point [50]. The intercept and growth factors are all correlated [50]. This linear-linear (straight line) piecewise LGM can be generalized to quadratic-quadratic piecewise LGM by adding a quadratic factor in each piece [50]. To separate the developmental stages or phases, a ‘knot’ or ‘transition point’ needs to be specified based on theory or empirical and graphical evidence, where the two phases of development are to be connected [50]. An example of the quadratic-quadratic two-piece LGM is presented in Figure 1 below.

Since the children were assessed biennially, the time scores were specified to reflect this measurement interval. For example, in the linear model, the time scores were 0, 2, 4, 6, 8, 10, and 12. In the quadratic model, the square of the time scores is specified for the quadratic factor. To assess how well each growth curve model fitted the data, the conventional cut-offs in approximate fit indices were adopted. The chi-square tests the exact fit hypothesis but is also very sensitive to minor misspecifications [51]; hence, greater emphasis is placed on the approximate fit indices. Specifically, CFI and TLI values above 0.95 and RMSEA and SRMR values below 0.05 and 0.08, respectively, were assumed to reflect good-fitting models [52]. To evaluate measurement invariance, the absolute differences (Δ) in CFI and RMSEA between two nested models were examined. Specifically, |ΔCFI| ≤ 0.01 and |ΔRMSEA| ≤ 0.015 were assumed to reflect measurement invariance [53]. Due to the ordinal nature of the item-level data, the WLSMV estimator was used for LMI testing. LGMs were estimated using robust maximum likelihood (MLR). Longitudinal sampling weights (including non-response adjustment), clustering, and stratification were incorporated in all inferential analyses to account for the stratified cluster sampling. All inferential analyses were run in Mplus 8.7 [54], whereas data merging was performed and descriptive statistics were calculated in Stata 17 [55].

### 2.5. Procedure

The LSAC child cohort began in 2004, when the children were 4 years old, and followed a research design that ensured the representativeness of the Australian population of children [31]. Participating families were visited biennially by trained interviewers to collect data for the main strand of the cohort survey [33]. In wave 1 (age 4), the interviewers administered a paper-based interview, whereas a computer-assisted interview was conducted from wave 2 onwards [31]. Prior to the full round of data collection, a pre-testing phase was conducted per wave to ensure the feasibility of the survey instruments [31]. After data collection, the data sets were confidentialized to reduce the risk of disclosure before being released for research purposes [31].

## 3. Results

### 3.1. Missing Data Analysis and Descriptive Statistics

There were 14.65% missing values across the seven internalizing scores across time. Using Little’s MCAR test [56], it was found that the missing data were not missing completely at random (MCAR). Adjusting for the child’s sex, it was found that Little’s MCAR test was not statistically significant (*p* > 0.05), suggesting that the missing values are dependent on other variables in the data set. Therefore, the missing at-random assumption was considered more appropriate. Missing values were handled using the default full-information maximum likelihood in Mplus. Next, weighted descriptive statistics (means, standard deviation, or percentages) were calculated for the sample and are presented in Table 1. 

### 3.2. Longitudinal Measurement Invariance of Internalising Symptoms

Before commencing with the LGM analyses, longitudinal measurement invariance analyses were conducted. The results of the invariance analyses are presented in Table 2. As shown in Table 2, both the emotional symptoms and the peer problems scale were strictly invariant over time, which permitted accurate comparisons of the observed variables’ means and standard deviations [51]. 

### 3.3. The Developmental Trajectory of Internalising Symptoms

Several latent growth curve models were estimated to determine the shape of the developmental trajectory. As shown in Table 3, a single-piece quadratic shape was better fitting than the simple linear model, which indicated that a nonlinear quadratic function would better approximate accelerating or decelerating individual trajectories. Following several configurations of two piecewise growth curves, it was confirmed (see Table 3) that the piecewise quadratic-quadratic growth curve model with a fixed transition knot at age 10 years fitted the data better (see Figure 1). This suggests that the development of internalizing symptoms is better characterized as a quadratic trajectory comprising two phases, namely, one between ages 4 and 10 years and another from age 10 up to age 16 years. The parameter estimates of the piecewise quadratic-quadratic LGM are presented in Supplemental Materials Table S1.

**Figure 2:**
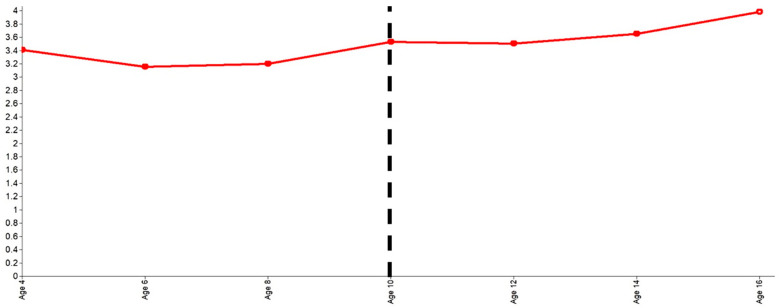
Quadratic-quadratic piecewise growth curve trajectory with the first piece stopping and the second piece beginning at age 10 years.

The average piecewise trajectory is shown in Figure 2. As shown in Table 4, the latent growth factor means, and their accompanied p-values give rise to the following interpretation. On average, the trajectory starts at age 4 with a score of 3.4 and then gradually declines, but by age 10 years, the trajectory is slightly becoming upwards concave. After the age of 10 years, the trajectory remained relatively stable (linear slope 2 mean *p* > 0.05), but by age 16 years, the trajectory slightly accelerated, taking an upward concave shape.

Finally, the correlations within phases and between phases are also important. As shown in Table 4, there were statistically significant correlations within each developmental phase but not between developmental phases. This suggests that the development of internalizing symptoms is largely independent between the two phases, and significant altering events occur in the transition to adolescence. 

### 3.4. Controlling for Standard Demographic Factors

As a robustness check, standard available demographic factors (see Section 2.3) were added as time-invariant predictors at age 4 years. The piecewise model’s fit slightly deteriorated with scaled χ^2^ (28) = 96.693, *p* < 0.002, CFI = 0.988, TLI = 0.974, RMSEA = 0.030, SRMR = 0.022. The statistical significance of the growth factors’ correlations remained invariant. Based on the regressions of the latent intercept and slope factors on the covariates, it was found that the standard demographic control variables had a marginal impact on the developmental trajectory already identified. 

Gender positively predicted the quadratic slope 2 (β = 0.140, *p* < 0.001) only, suggesting that females had greater curvature (more upward trajectory) in late adolescence. The responding parent’s income negatively predicted the intercept (β = −0.085, *p* < 0.001), suggesting a lower initial score at age 4 years, but it did not predict the slopes. In the same vein, the responding parent’s highest educational qualification predicted the intercept (β = −0.059, *p* < 0.05) but did not predict the slopes. Married status (versus not married) predicted lower symptom scores at age 4 years (β = −0.109, *p* < 0.05) but did not predict the slopes. Whether the parent was attending a religious service was generally not a statistically significant predictor of the intercept or the slope factors (*p* > 0.05). Having more siblings marginally predicted lower internalizing scores at the baseline (β = −0.062, *p* < 0.05) and a slower linear growth (β = −0.085, *p* < 0.05) but did not predict the quadratic slopes or the second piece’s linear slope. Detailed statistical information about the control variables is provided in the Supplemental Materials Table S2.

Overall, these statistics suggest that the piecewise development of internalizing symptoms is largely independent of standard demographic time-invariant covariates at age 4 years.

## 4. Discussion

The present study examined the developmental continuity and discontinuity in children’s internalizing mental health symptoms across early childhood and up to late adolescence. The current study contributes to the international literature in three important ways. First, the study confirms that child internalizing mental health development is not only a continuous process but also characterized by discontinuity that is especially identified at the onset of early adolescence. Second, the study revealed that the development of internalizing symptoms was characterized by two phases of growth that appeared to be largely independent. Last but not least, the findings illustrate that the trajectory of internalizing symptoms did not shift, adjusting for standard demographic covariates. 

To address the first research question, several latent growth curve models (LGM) were estimated and compared. The results of the LGM modelling revealed a clear preference for the quadratic growth function over the linear growth function in internalizing symptoms’ development across childhood and up to late adolescence. This finding complies with previous evidence suggesting that internalizing symptoms might be better represented by a quadratic growth function rather than the simpler linear growth function [1,15]. Additionally, this result contradicts previous evidence that utilized linear functions to approximate growth in internalizing symptoms [2,14]. Hence, H1 was supported. The strong evidence in favor of a quadratic function suggests that there is a curvature in children’s internalizing development over time, and perhaps researchers should attempt to gather at least four waves of repeated measures to appropriately model the internalizing symptoms’ development.

Following up on the quadratic LGM, I ran several other more complicated piecewise LGMs to (dis-)confirm whether internalizing symptoms’ development was better approximated by piecewise growth. Piecewise LGM is an appropriate modeling choice since it can separate the development into two phases, whereby each phase is characterized by continuity, but there might be discontinuity across phases [50]. In contrast to previous evidence suggesting the existence of linear-linear piecewise growth in internalizing symptoms [5,6,19], the current study provides some initial evidence in favor of quadratic-quadratic piecewise growth starting at age 10 years. Overall, the finding of a two-part nonlinear growth trajectory is highly innovative and challenges previous evidence on the continuity of the development of children’s internalizing mental health [2,14,39]. Hence, H2 was supported. 

The age of 10 years is usually assumed to reflect the beginning of early adolescence [24,25], which closely ties in with the significance of this developmental period for later positive outcomes [57]. Adolescence has been characterized by many developmental milestones that adolescents have to deal with [22,23], and therefore, there is theoretical support in favor of specifying a ‘transition point’ at this specific age. Hence, H3 was supported. Nevertheless, the parameters of the two-piece quadratic-quadratic LGM suggested that the correlations across the two phases of internalizing symptoms’ development did not reach statistical significance, indicating that initial declines in internalizing symptoms during the first phase were not necessarily linked with the concave increase in the second phase in the adolescent years. This is an important finding since theory suggests that the positive effects of earlier development might be reversed or completely erased by critical turning points in later development [16], which appears to be the case here. Adolescence has been noted in the past to be a period in life when internalizing symptoms become more pronounced [10,23], and the current findings seem to partially support such claims. Yet, it should be mentioned that for a short while, between ages 10 and 14 years, the development of internalizing symptoms was more stable, whereas it spiked upward between 14 and 16 years. This suggests the need for greater support for adolescents in the transition from mid-adolescence to later adolescence. 

### 4.1. Strengths and Limitations

Overall, the current study has several strengths, such as the representative sample, the long-term design, the equal measurement intervals, and the robust screening measures of child and adolescent mental health. However, some limitations should be outlined. For instance, the SDQ is not a measure of a specific mental health disorder; rather, it is a generalized screening measure that, nonetheless, has good psychometric properties. Another limitation of the current study is the absence of further measurement occasions after the age of 16 years, which prohibits the examination of further piecewise growth patterns in emerging adulthood. Finally, the parent-reported nature of the screening measure might consist of a limitation, but this is very common in large-scale cohort studies in the community. 

### 4.2. Future Directions

Several future directions arise from the current study. Future research could focus on piecewise growth in internalizing mental health symptoms in even longer spans to gain greater insights. Further, it is important to examine what types of time-invariant covariates, beyond gender and income, and what time-varying covariates influence the shape of the piecewise trajectory. Additionally, replication of the current piecewise trajectory findings is recommended with other representative samples. Finally, future research can focus on more time-intensive designs to estimate the internalizing symptoms’ development in a momentary fashion. 

## 5. Conclusions

In conclusion, the research findings also address an outstanding issue regarding the discontinuity in children’s internalizing mental health symptoms’ development. The trajectory of internalizing symptoms was better characterized as quadratic-quadratic piecewise, with one phase stopping at age 10 and the other beginning after 10 years old. The two phases of internalizing symptoms’ development were largely unrelated, suggesting that there is a significant discontinuity in children’s mental health development. The onset of early adolescence at 10 years appeared to be a significant transitioning point that completely reversed children’s internalizing symptoms’ scores. More emphasis and support are needed during the transition from mid-adolescence to later adolescence (ages 14 to 16) since there was an upward increase in internalizing symptoms at that point. 

## Figures and Tables

**Table 1:** Sample’s descriptive statistics (weighted by sampling weight).

Variable	Descriptive Statistic (Mean, SD or %)
Responding parent’s sex (ref: Male)	
Female	97.17%
Sex (ref: Male)	
Female	49.10%
Parent’s income (ref: lowest category)	
$500–$999 pw $26,000–$51,999 per year	25.6%
$1000–$1999 pw $52,000–$103,999 per year	7.98%
$2000 or more per week $104,000 or more per year	1.01%
Language spoken at home by child (ref: not English)	
English	90.8%
Highest parental education (ref: not university)	
University-level education	49.90%
Parent’s marital status (ref: not married)	
Married	84.01%
Parent attending religious services (ref: no)	
Yes	35.49%
Number of siblings in household	M = 1.48, SD = 0.99
Internalizing symptoms (age 4)	M = 3.39, SD = 2.69
Internalizing symptoms (age 6)	M = 3.20, SD = 2.78
Internalizing symptoms (age 8)	M = 3.13, SD = 2.90
Internalizing symptoms (age 10)	M = 3.52, SD = 3.19
Internalizing symptoms (age 12)	M = 3.51, SD = 3.12
Internalizing symptoms (age 14)	M = 3.59, SD = 3.16
Internalizing symptoms (age 16)	M = 3.95, SD = 3.45

*Note*: Ordinal variables are presented as percentages and interval variables are presented as means (M) and standard deviations (SD); pw: per week; $ Australian dollar; Weighted descriptive statistics are presented.

**Table 2:** Differences in approximate fit measures of longitudinal measurement invariance analyses from age 4 to age 17 (single-group approach).

Invariance Level	Scaled χ^2^ (df)	CFI	|ΔCFI|	RMSEA	|ΔRMSEA|
	Emotional symptoms				
Configural	1599.634 (529) ***	0.964		0.027	
Metric	1740.096 (553) ***	0.960	0.004	0.028	0.001
Scalar	1696.709 (577) ***	0.962	0.002	0.026	0.002
Strict	2022.697 (607) ***	0.952	0.010	0.029	0.003
	Peer problems				
Configural	1141.673 (529) ***	0.970		0.020	
Metric	1230.926 (553) ***	0.967	0.003	0.021	0.001
Scalar	1339.942 (577) ***	0.963	0.004	0.022	0.001
Strict	1516.078 (607) ***	0.956	0.007	0.023	0.001

*Note*: n = 2792; *** *p* < 0.001; df: degrees of freedom; CFI: Comparative Fit Index; |ΔCFI|: Difference in CFI; RMSEA: Root mean square error of approximation; |ΔRMSEA|: Difference in RMSEA.

**Table 3:** Growth Curve Models’ Fit Indices.

Trajectory Shape Specification	Scaled χ^2^ (df)	CFI	TLI	RMSEA	SRMR
Linear	313.449 (23) ***	0.938	0.943	0.067	0.066
Quadratic	139.758 (19) ***	0.974	0.972	0.048	0.028
Piecewise linear-linear knot at age 8	127.564 (19) ***	0.977	0.974	0.045	0.028
Piecewise linear-quadratic knot at age 8	60.123 (14) ***	0.990	0.985	0.034	0.019
Piecewise linear-linear knot at age 10	146.586 (19) ***	0.973	0.970	0.049	0.030
Piecewise linear-quadratic knot at age 10	109.475 (14) ***	0.980	0.969	0.049	0.026
**Piecewise quadratic-quadratic knot at age 10**	**11.902 (8) ns**	**0.999**	**0.998**	**0.013**	**0.009**
Piecewise linear-linear knot at age 12	175.730 (19) ***	0.967	0.963	0.054	0.042
Piecewise quadratic-linear knot at age 12	66.419 (14) ***	0.989	0.983	0.037	0.020

*Note*: n = 2792; *** *p* < 0.001; ns: not statistically significant; boldfaced values indicate best fitting model; df: degrees of freedom; CFI: Comparative fit index; RMSEA: Root mean square error of approximation; TLI: Tucker-Lewis Index; SRMR: Standardized root mean residual; linear-linear describes a model with two linear slopes; quadratic-linear describes a model with one quadratic slope in addition to a linear slope in the first phase and one linear slope in the second phase; linear-quadratic describes a model with one linear slope in the first phase and one quadratic slope in addition to a linear slope in the second phase; the knot is the fixed transition time point when one phase stops and the other phase begins, and both phases are joined.

**Table 4:** Key parameter estimates of the quadratic-quadratic piecewise growth curve model.

Path Specification	Parameter Estimate (S.E.)	Two-Tailed *p*-Value
Correlations		
Linear slope 1—Intercept	−0.379 (0.098)	0.000
Quadratic slope 1—Intercept	0.315 (0.106)	0.003
Linear slope 1 –Quadratic slope 1	−0.922 (0.017)	0.000
Linear slope 2—Intercept	−0.158 (0.067)	0.019
Linear slope 1—Linear slope 2	−0.250 (0.131)	0.057
Linear slope 2—Quadratic slope 1	0.258 (0.204)	0.205
Quadratic slope 2—Intercept	0.070 (0.056)	0.194
Quadratic slope 2—Linear slope 1	0.157 (0.088)	0.072
Quadratic slope 1—Quadratic slope 2	−0.179 (0.129)	0.165
Quadratic slope 2—Linear slope 2	−0.852 (0.027)	0.000
Latent means (unstandardized)		
Intercept	3.407 (0.067)	0.000
Linear slope 1	−0.198 (0.035)	0.000
Quadratic slope 1	0.036 (0.005)	0.000
Linear slope 2	−0.059 (0.034)	0.086
Quadratic slope 2	0.022 (0.006)	0.000
Latent variances (unstandardized)		
Intercept	5.247 (0.564)	0.000
Linear slope 1	0.889 (0.177)	0.000
Quadratic slope 1	0.016 (0.003)	0.000
Linear slope 2	0.412 (0.113)	0.000
Quadratic slope 2	0.017 (0.003)	0.000

*Note*: INTt: Internalizing symptoms at time t; Slope 2 refers to the second piece of the trajectory (adolescence- age 10 years onwards); Slope 1 refers to the first piece of the trajectory (up to 10 years); S.E.: Standard error.

## Data Availability

The data are available through the Australian Data Archive at https://doi.org/10.26193/QR4L6Q (accessed on 8 October 2023).

## Supplementary Materials

The following supporting information can be downloaded at: https://www.mdpi.com/article/10.3390/ejihpe14080159/s1, Table S1: Latent Growth Curve Factor Parameters; Table S2: Linear regression effects of the control variables on the intercept (i.e., baseline scores), the linear slope 1 (ages 4 to 8), quadratic slope 1 (ages 4 to 8), linear slope 2 (ages 10 to 16), and quadratic slope 2 (ages 10 to 16)
