# Supplementary material for: Development of Internalizing Mental Health Symptoms from Early Childhood to Late Adolescence
Source: Eur J Investig Health Psychol Educ. 2024 Aug 18;14(8):2404–16. doi: 10.3390/ejihpe14080159 (PMC11353613; doi:10.3390/ejihpe14080159)
Supplement: Supplementary file 1 [file ejihpe-14-00159-s001.zip › ejihpe-3106515-supplementary.pdf]

Supplemental Materials for the Article Entitled "Development of internalizing mental health symptoms from early childhood to late adolescence"

Table S1. Latent Growth Curve Factor Parameters

Intercept loadings	Parameter estimate	Two-tailed p-value
INT1 (age 4 years)	1	.999
INT2 (age 6 years)	1	.999
INT3 (age 8 years)	1	.999
INT4 (age 10 years)	1	.999
INT5 (age 12 years)	1	.999
INT6 (age 14 years)	1	.999
INT7 (age 16 years)	1	.999
Linear slope 1 loadings		
INT1 (age 4 years)	0	.999
INT2 (age 6 years)	2	.999
INT3 (age 8 years)	4	.999
INT4 (age 10 years)	6	.999
INT5 (age 12 years)	6	.999
INT6 (age 14 years)	6	.999
INT7 (age 16 years)	6	.999
Quadratic slope 1 loadings		
INT1 (age 4 years)	0	.999
INT2 (age 6 years)	4	.999
INT3 (age 8 years)	16	.999
INT4 (age 10 years)	36	.999
INT5 (age 12 years)	36	.999
INT6 (age 14 years)	36	.999
INT7 (age 16 years)	36	.999
Linear slope 2 loadings		
INT1 (age 4 years)	0	.999
INT2 (age 6 years)	0	.999
INT3 (age 8 years)	0	.999
INT4 (age 10 years)	0	.999
INT5 (age 12 years)	2	.999
INT6 (age 14 years)	4	.999
INT7 (age 16 years)	6	.999
Quadratic slope 2 loadings		
INT1 (age 4 years)	0	.999
INT2 (age 6 years)	0	.999
INT3 (age 8 years)	0	.999
INT4 (age 10 years)	0	.999
INT5 (age 12 years)	4	.999
INT6 (age 14 years)	16	.999
INT7 (age 16 years)	36	.999
Residual variances (standardised)		
INT1 (age 4 years)	.278 (.071)	.000
INT2 (age 6 years)	.386 (.023)	.000
INT3 (age 8 years)	.305 (.025)	.000
INT4 (age 10 years)	.295 (.036)	.000
INT5 (age 12 years)	.295 (.019)	.000
INT6 (age 14 years)	.317 (.020)	.000
INT7 (age 16 years)	.103 (.064)	.108
R-squared		

INT1 (age 4 years)	.722	.000
INT2 (age 6 years)	.614	.000
INT3 (age 8 years)	.695	.000
INT4 (age 10 years)	.705	.000
INT5 (age 12 years)	.705	.000
INT6 (age 14 years)	.683	.000
INT7 (age 16 years)	.897	.000

Note: .999 means that the parameter was fixed for model identification (standard for piecewise growth curve modelling)

Table S2: Linear regression effects of the control variables on the intercept (i.e., baseline scores), the linear slope 1 (ages 4 to 8), quadratic slope 1 (ages 4 to 8), linear slope 2 (ages 10 to 16), and quadratic slope 2 (ages 10 to 16)

	Beta coef.	S.E.	Beta/S.E.	P-value
I ON				
Female	-0.036	0.026	-1.411	0.158
Income	-0.085	0.027	-3.139	0.002
Education	-0.059	0.030	-1.973	0.049
Marital status	-0.109	0.033	-3.287	0.001
Religious service	0.018	0.025	0.714	0.475
Number of siblings	-0.062	0.031	-2.037	0.042
S1 ON				
Female	0.019	0.037	0.505	0.614
Income	0.069	0.039	1.785	0.074
Education	0.027	0.045	0.598	0.550
Marital status	-0.070	0.043	-1.615	0.106
Religious service	0.014	0.035	0.391	0.696
Number of siblings	-0.085	0.040	-2.097	0.036
S2 ON				
Female	-0.039	0.055	-0.703	0.482
Income	0.012	0.055	0.223	0.823
Education	-0.039	0.061	-0.647	0.517
Marital status	-0.002	0.060	-0.029	0.977
Religious service	0.057	0.050	1.142	0.253
Number of siblings	0.018	0.057	0.320	0.749
Q1 ON				
Female	-0.016	0.044	-0.361	0.718
Income	-0.078	0.043	-1.810	0.070
Education	-0.063	0.055	-1.143	0.253
Marital status	0.053	0.052	1.018	0.309
Religious service	-0.052	0.043	-1.192	0.233
Number of siblings	0.074	0.048	1.552	0.121
Q2 ON				
Female	0.140	0.048	2.908	0.004
Income	-0.019	0.043	-0.447	0.655
Education	0.039	0.051	0.766	0.443
Marital status	-0.017	0.050	-0.347	0.728
Religious service	-0.067	0.043	-1.543	0.123
Number of siblings	-0.006	0.046	-0.138	0.890

Note: I: Intercept; S1: Linear slope 1; S2: Linear slope 2; Q1: Quadratic slope 1; Q2: Quadratic slope 2; Beta: Standardized regression coefficient; S.E.: Standard error; ON: means regressed on
